# A Potential Diagnostic and Prognostic Biomarker TMEM176B and Its Relationship With Immune Infiltration in Skin Cutaneous Melanoma

**DOI:** 10.3389/fcell.2022.859958

**Published:** 2022-03-23

**Authors:** Linlan Jiang, Yanyin Yang, Fangming Liu, Mingyue Ma, Jie Gao, Lu Sun, Yuwen Chen, Zan Shen, Duojiao Wu

**Affiliations:** ^1^ Department of Oncology, Affiliated Sixth People’s Hospital, Shanghai Jiaotong University, Shanghai, China; ^2^ Department of Endocrinology and Metabolism, Zhongshan Hospital, Key Laboratory of Metabolism and Molecular Medicine, the Ministry of Education, Fudan University, Shanghai, China; ^3^ Institute of Clinical Science, Zhongshan Hospital, Fudan University, Shanghai, China; ^4^ Jinshan Hospital Center for Tumor Diagnosis and Therapy, Jinshan Hospital, Fudan University, Shanghai, China

**Keywords:** TMEM176B, cancer, CD8^+^ T cell, tumor immunology, SKCM

## Abstract

**Background:** Melanoma is a highly malignant and aggressive tumor. The search for new and effective biomarkers facilitates early diagnosis and treatment, ultimately improving the prognosis of melanoma patients. Although the transmembrane protein TMEM176B has been linked to a number of cancers, its role in cancer immunity remains unknown.

**Methods:** Expression levels of TMEM176B in normal tissues and several cancers, including Skin Cutaneous Melanoma (SKCM), were collected from TCGA and GTEx. We used Receiver operating characteristic and Kaplan–Meier survival curves and performed regression analysis to elucidate the link between TMEM176B and clinicopathological features of SKCM in order to determine the prognostic significance of TMEM176B in SKCM. We then used the GEPIA and STRING websites to search for proteins and associated top genes that may interact with TMEM176B and enriched them for analysis. The link between TMEM176B and immune cells infiltration was then investigated using TIMER, CIBERSORT algorithm and GSVA package of R (v3.6.3). Finally, animal tests were conducted to confirm the expression of *Tmem176b* and its influence on T-cell immune infiltration.

**Results:** *TMEM176B* expression was considerably elevated in SKCM compared to normal tissues. Particularly, *TMEM176B* expression was also linked to pathological stage, tumor ulceration and radiation therapy. Patients with elevated *TMEM176B* expression had a better prognosis, according to the survival analysis. The majority of tumor infiltrating lymphocytes (TILs) especially T cells in SKCM was positively linked with *TMEM176B* expression. Our animal experiments also verified that the T-cell infiltration was significantly inhibited in local melanoma tissue of *Tmem176b* knockout mice. At the same time deleting *Tmem176b* accelerated tumor progress and impaired T cells effector function.

**Conclusion:** Upregulated expression of TMEM176B in SKCM is associated with a better prognosis and it has the potential to serve as a diagnostic and prognostic marker for the disease. It may serve as a target for SKCM immunotherapy by regulating CD8^+^ T cells although it requires more evidence.

## Introduction

Melanoma, which is aggressive, is a malignant tumor that develops from melanocytes (Schadendorf et al., 2015; Shain and Bastian, 2016; Lin and Fisher, 2017). Cutaneous melanoma is most common in Western countries, but the global incidence has gradually increased in recent years (Hollestein et al., 2012). Ultraviolet light (UV) radiation is an important pathogenic factor in the development of melanoma (Schadendorf et al., 2015; Michielin et al., 2019). Early diagnosis is made easier by general clinical and pathological aspects due to the specific color characteristics of melanoma and the fact that it occurs on the skin surface, although diagnostic hurdles still exist (Schadendorf et al., 2015). Patients with advanced metastases have a poor prognosis, despite the fact that early-stage tumors can be surgically excised with a high cure rate (Lin and Fisher, 2017). As a result, new diagnostic and prognostic biomarkers are needed to increase the survival rate of melanoma patients.

To achieve complete control of tumors, the antitumor effector cells in the immune system must coordinate with each other to overcome tumor immune evasion (Huntington et al., 2020). T cells are one of the most important anti-tumor effector cells because they may directly attack cancer cells. Immune checkpoint therapy targeting T cells, for instance, cytotoxic T lymphocyte-associated antigen 4 (CTLA4) and programmed cell death protein 1 (PD1) has shown promising results in patients with melanoma in recent years, but only some tumor types have benefited, the degree of tumor infiltration and immune cell activation, particularly CD8^+^ T cells, are both factors that influence immune checkpoint therapy efficacy (Kim et al., 2020). Exploring the immune phenotype and interactions in the tumor microenvironment is therefore critical for discovering new immunotherapeutic targets in melanoma.

Some transmembrane proteins have been found to be overexpressed in tumors and to be linked to prognosis, suggesting that they could be useful targets for immunotherapy (Choi et al., 2007; Tomimaru et al., 2015). TMEM176B (Transmembrane protein 176B), which was formerly known as TORID (Tolerance-related and Inducible Transcript) (Louvet et al., 2005), is a gene that is, highly overexpressed in tolerance allografts (Viklicky et al., 2013). Its human homolog was originally found to be expressed in a subset of lung fibroblasts, the mouse homolog Clast1 was upregulated after B cells were activated by CD40 ligand (Lurton et al., 1999). TMEM176B, like its paralog TMEM176A (Transmembrane protein 176A), has four transmembrane domains and belongs to the MS4A family (Eon Kuek et al., 2016). It is mostly expressed in the lungs, kidneys, lymph nodes, and spleen, among different immune cell subtypes, it has been observed that the high expression of *Tmem176b* and *Tmem176a* in BMDCs (Bone Marrow-Derived Dendritic Cells) and cDCs (Classic Dendritic Cells), and is associated with the immature state of DCs (Dendritic Cells) (Condamine et al., 2010).

The expression level of TMEM176B was higher in gastric cancer tissues than in normal tissues, and according to survival analysis, higher expression levels were related to poorer prognosis (Sun et al., 2018). The expression of TMEM176B in lymphoma and its control tissues also differed significantly (Cuajungco et al., 2012; Ma and Li, 2021). The expression of TMEM176B is up-regulated in the tumor blood vessels of human renal cell carcinoma specimens, suggesting that it is implicated in tumor angiogenesis and could be a target of anti-angiogenesis therapy for cancer patients (Otsubo et al., 2014). However, the role of TMEM176B in cancers has yet to be determined, or we do not yet have a complete grasp of its function in malignancies.

In addition, the correlation between TMEM176B and tumor immunity and its role in melanoma is less reported. *Tmem176b* and its homolog *Tmem176a* has been found to be highly expressed in CD4^+^ Th17 cells (Drujont et al., 2016), this study also found that these two molecules play the same ion channel function and are co-localized near the Golgi apparatus (Drujont et al., 2016). The expression of some costimulatory molecules in T cells can be inhibited by transfection of *Tmem176b* into immature dendritic cells (Louvet et al., 2005). It revealed that suppressing inflammasomes by knocking down *Tmem176b* in mice or utilizing TMEM176B inhibitors can improve the anti-tumor effect of CD8^+^ T and the efficacy of anti-CTLA-4 and anti-PD1 therapy (Segovia et al., 2019). However, cancer is a very heterogeneous disease and melanoma has its unique immune microenvironment. It is unknown the role of TMEM176B in regulating CD8^+^ T cells biology and the course of melanoma.

We explored the expression of TMEM176B in SKCM and its relationship with tumor patients’ prognosis and immune infiltration. Furthermore, we found the regulatory effect of TMEM176B on tumor-infiltrating CD8^+^ T cells. Therefore, TMEM176B is a potential diagnostic or prognostic biomarker for melanoma.

## Methods

### Gene Expression Analysis

We utilized R package “ggplot2” to analysis the differential expression of *TMEM176B* in various malignancies or cancer subtypes. Extraction of SKCM in TCGA and corresponding normal tissue data in GTEx has been analyzed for differential expression of *TMEM176B* in cutaneous melanoma. Click “repository” in TCGA database and make the following settings: under File directory (select “Gene Expression Quantification” under Data Category module, select “HTseq-FPKM” under Workflow Type module); under Cases (under Primary Site module, select “skin,” and under Project, select “TCGA-SKCM”); under Cases (select “skin” under the Primary Site module and “TCGA-SKCM” under Project) to get the clinical information of SKCM.

### Survival Prognosis Analysis

R package “survminer” and “survival” were utilized to analyze the prognostic value of the expression level of *TMEM176B* in different tumors. We collected the data from the TCGA database (Liu et al., 2018) and compared the overall survival (OS) of cancer patients separated by the median expression level of *TMEM176B*. The “pROC” package and the “ggplot2” package were used to extract and analyze the SKCM data of TCGA and the corresponding normal tissue data in GTEx to obtain the receiver operating characteristic (ROC) curves.

### Cox Regression

We performed Cox regression analysis to identify the association between SKCM disease characteristics and the expression level of *TMEM176B*.

### Survival Analysis in PrognoScan

In PrognoScan, the association between TMEM176B expression and prognosis of cancer patients was analyzed, including distant metastasis-free survival (DMFS), disease-free survival (DFS), relapse-free survival (RFS), OS, and distant recurrence-free survival (DRFS).

### Relationship Between TMEM176B Expression and Immune Cell Infiltration

TIMER is a fully functional public platform for analyzing of immune infiltration in tumors. Correlations between *TMEM176B* expression and immune infiltration levels in more than 30 different cancer types in TCGA were obtained in the gene module of TIMER. We also investigated the connection between tumor purity and TMEM176B expression. And we utilized R package “GSVA” to obtain the link among TMEM176B and other immunocytes (Bindea et al., 2013). Next “CIBERSORT algorithm” was used to explore the immune infiltration between high- and low-expression with *TMEM176B* to obtain enriched immunocytes between the two groups.

### TMEM176B-Related Gene Enrichment Analysis and Single Gene Co-Expression Analysis

We acquired the top 100 genes associated with *TMEM176B* in SKCM cases using the “Similar Gene Detection” module of GEPIA2 and performed correlation analysis on the top 5 genes using “Correlation Analysis” in GEPIA2. We then obtained protein networks that may interact with TMEM176B in the STRING database. In addition, we subjected the 100 genes obtained in GEPIA2 to GO and KEGG pathway analysis using the “clusterProfiler” and “ggplot2” R packages.

R package “ggplot2” was used to determine the link between the expression level of TMEM176B in SKCM and other genes, including CD274, CTLA4, GZMA, GAMB, IFNG, PRF1, and so on.

### Animals

Aged 6–8 weeks *Tmem176b* ^−/−^ mice and C57BL/6J mice were purchased from GemPharmatech Co. Ltd. and Shanghai Jie Si Jie Laboratory Animal Co. Ltd., respectively.

### Cell Culture

B16-OVA melanoma cells were cultured in DMEM medium, which contains 10% fetal bovine serum (FBS) and 1% penicillin/streptomycin.

### Tumor Model

We injected 1 × 10^6^ B16-OVA cells into the right flank of WT or *Tmem176b* ^−/−^ mice subcutaneously and used vernier calipers to measure tumor volume every 1–2 days. On the 14th day, the mice were euthanized and their tumor tissues, spleen, lymph nodes, and peripheral blood were taken for further research.

### Flow Cytometry

Cells were stained with CD3 (BioLegend, clone:17A2), CD4 (BioLegend, clone: GK1.5), CD8 (BioLegend, clone:53-6.7), GZMB (Biolegend, clone: QA16A02), IFN-γ (Biolegend, clone: XMG1.2), Ki-67 (BioLegend, clone: 16A8), PD-1(Biolegend, clone:29F.1A12), PD-L1 (BioLegend, clone:10F.9G2), CD44 (Biolegend, clone: IM7), CD62L (BioLegend, clone: MEL-14), CD25(BioLegend, clone: 3C7), CD69(BioLegend, H1.2F3), BCL2 (Cell Signaling Technology, clone: 124), PRF(BioLegend, clone: S16009A). For intracellular staining, cells were given PMA/ionomycin (BioLegend, Cat. No. 423303) re-stimulation for 4–5 h, fixed, permeabilized, and then stained. FACS analysis was performed on a BD FACS Aria III flow cytometer and analyzed by FlowJo V.10 software. BD FACS Aria III flow cytometer was used to perform FACS analysis and data analysis was performed in FlowJo V.10 software.

### Statistical Analysis

Graphpad Prism (v8) and R language version (3.6.3) were utilized for designing figures and statistical analysis. Wilcoxon rank sum test and Student’s t tests were used to compare the expression of TMEM176B in different groups. When *p* values were less than 0.05, differences were considered significant.

## Results

### Clinical Characteristics of Patients

We extracted clinical information from the TCGA, including TNM stage, pathologic stage, age, race, weight, gender and whether or not they had received radiation. Supplementary Table S1 summarizes all the information. The table manifested that the expression of *TMEM176B* is closely related to the TNM stage of SKCM, the pathological stage and radiation therapy.

### The Expression of TMEM176B in SKCM Was Increased

**FIGURE 1:**
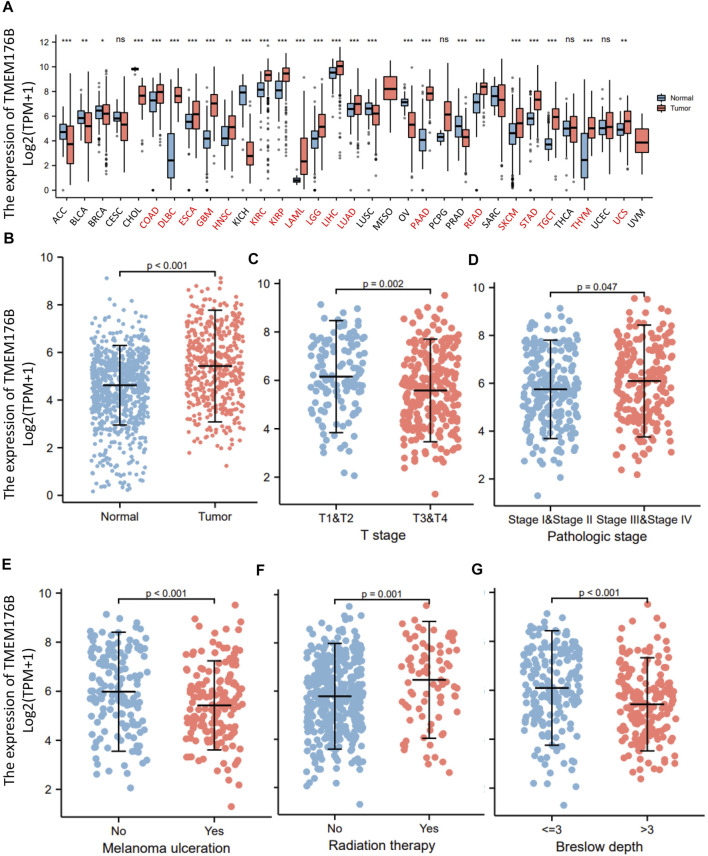
The expression of *TMEM176B* in malignancies. **(A)** The expression status of the *TMEM176B* across various cancer types. *p < 0.05; **p < 0.01; ***p < 0.001. **(B)** The expression level of *TMEM176B* in SKCM was significantly increased compared to the normal tissue (*p* < 0.001). **(C–G)** Association between *TMEM176B* and several clinical features in SKCM.

We demonstrated the expression status of *TMEM176B* across various types of cancer. *TMEM176B* expression was considerably enhanced in some malignancies, as demonstrated in Figure 1A, which further demonstrates that heterogeneity is an important attribute of cancer and a major contributor to tumor progression. Compared to normal tissue, *TMEM176B* expression was higher in SKCM (*p* <0.001) (Figure 1B). The correlation analysis revealed that there was a statistical difference between *TMEM176B* and T stage, pathologic stage (*p* <0.05), and melanoma ulceration, radiation therapy, breslow depth (*p* <0.001) (Figures 1C–G).

### The Prognostic Value of TMEM176B in SKCM

**FIGURE 2:**
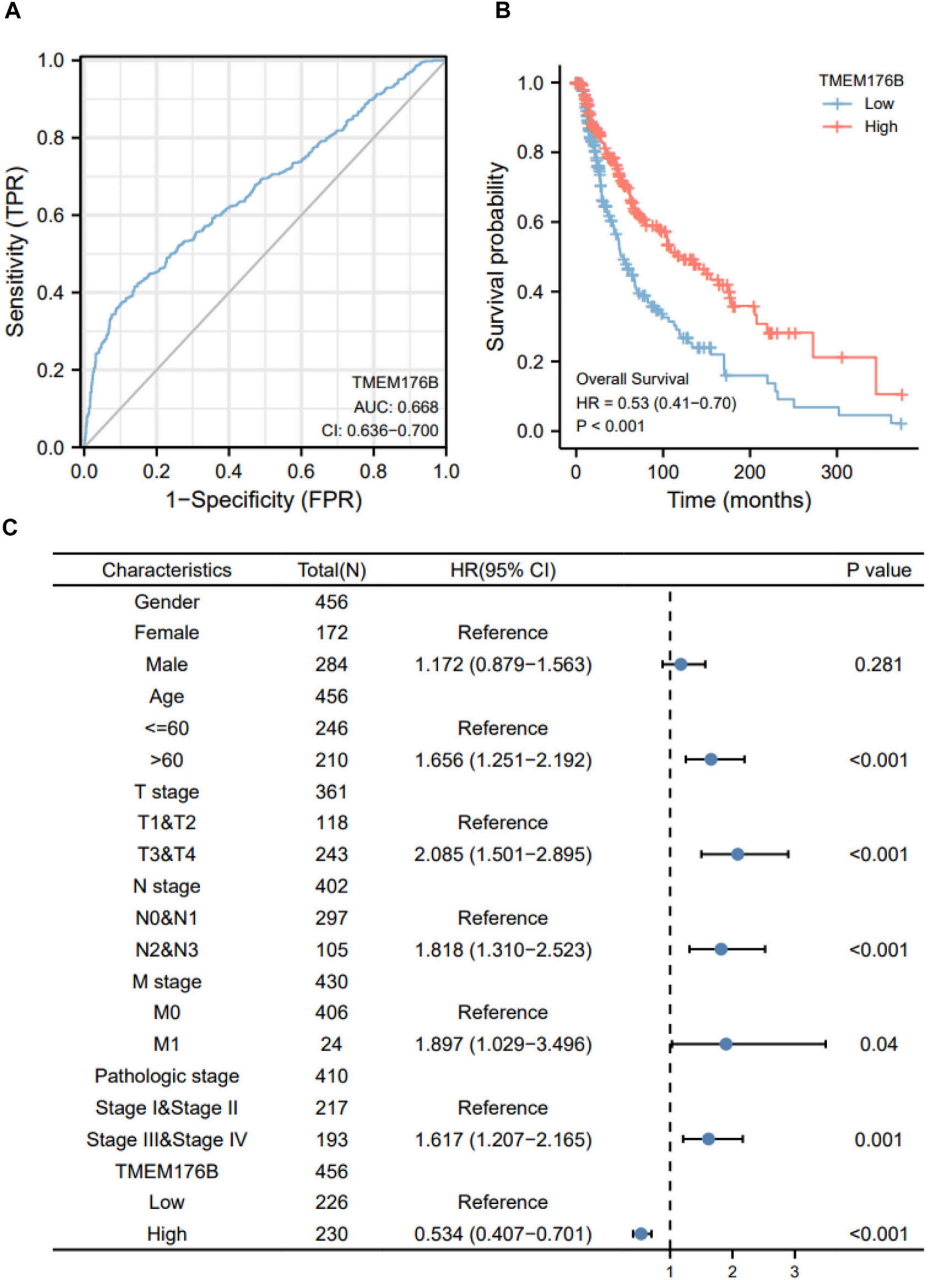
The prognostic value of *TMEM176B*. **(A)** ROC analysis showed that *TMEM176B* was able to identify tumor and normal tissue, the AUC was 0.668. **(B)** Survival evaluation indicators OS, *via* the expression level of the *TMEM176B* gene in SKCM. **(C)** Univariate Cox regression analysis of *TMEM176B* and clinicopathological variables with OS in SKCM.

Since the significant differential expression within tumor and normal tissues, we investigated the effect of *TMEM176B* expression on the prognosis of tumor patients. Cancer cases were separated into high- and low-expression groups based on *TMEM176B* median expression levels to evaluate how *TMEM176B* expression influences prognosis of patients in SKCM. The area under the curve can be derived from the ROC curve as 0.668 (Figure 2A). High *TMEM176B* level is linked with a better prognosis, according to the Kaplan-Meier survival analysis (Figure 2B). We also summarized in Supplementary Figures 1, 2 that the expression level of *TMEM176B* is closely associated with the prognosis of patients with various malignancies. We next combined clinical and pathological data, and analyzed the association between *TMEM176B* expression and a number of clinical parameters in SKCM patients. For OS, univariate Cox regression showed that *TMEM176B* played a detrimental role in patients with SKCM with the following characteristics: age >60 (*n* = 210, HR = 1.656, 95% CI from 1.251 to 2.192, *p* < 0.001), T stage 3 and 4 (*n* = 243, HR = 2.085, 95% CI from 1.501 to 2.895, *p* < 0.001), N stage 2 and 3 (*n* = 105, HR = 1.818, 95% CI from 1.310 to 2.523, *p* < 0.001), M stage 1 (*n* = 24, HR = 1.897, 95% CI from 1.029 to 3.496, *p* < 0.05), and Pathologic stage III and IV (*n* = 193, HR = 1.617, 95% CI from 1.207 to 2.165, *p* = 0.001) (Figure 2C). It is clear from this that TMEM176B plays an important role and serves as a reliable prognostic marker in SKCM.

### TMEM176B-Related Gene Enrichment Analysis

**FIGURE 3:**
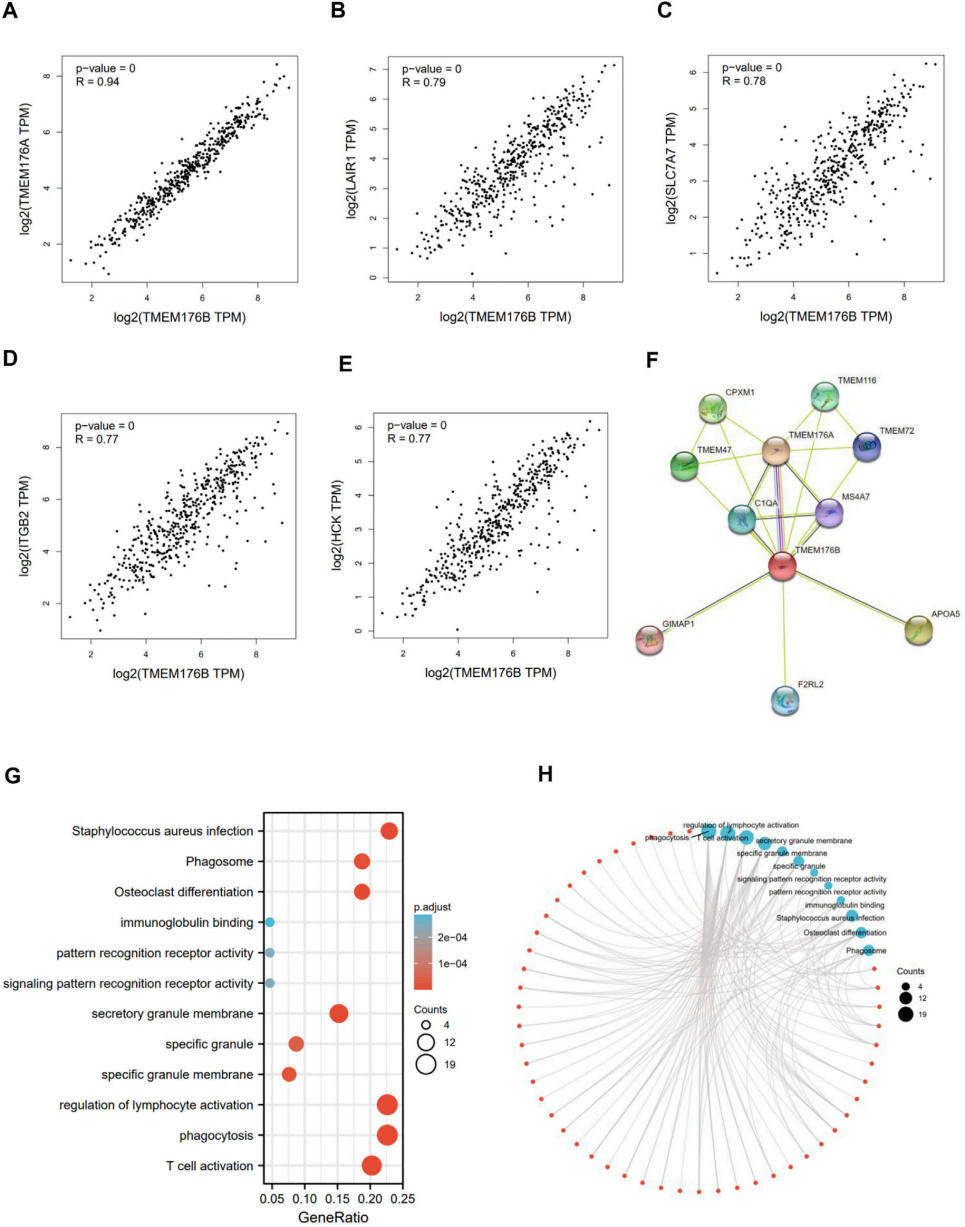
TMEM176B-related gene enrichment analysis. **(A–E)** Using GEPIA2, we identified the top 100 genes related with *TMEM176B* in SKCM and examined the expression connection between *TMEM176B* and five genes, including*TMEM176A*, *LAIR1*, *SLC7A7*, *ITGB2*, and *HCK*. **(F)** Protein networks that may interact with TMEM176B, data from STRING. **(G,H)** Enrichment analysis of *TMEM176B*-related genes.

We screened for proteins or related genes that might bind to TMEM176B in order to learn more about how it plays a role in carcinogenesis. GEPIA2 was used to identify the top 100 genes with the strongest relationship to *TMEM176B* in SKCM. The expression level of *TMEM176B* is positively linked with *TMEM176A* (Transmembrane Protein 176A) (R = 0.94), *LAIR1* (Leukocyte Associated Immunoglobulin Like Receptor 1) (R = 0.79), *SLC7A7* (Solute Carrier Family 7 Member 7) (R = 0.78), *ITGB2* (Integrin Subunit Beta 2) (R = 0.77), *HCK* (HCK Proto-Oncogene, Src Family Tyrosine Kinase) (R = 0.77), as illustrated in Figures 3A–E. The STRING program was also used to generate a network of proteins that interact with TMEM176B (Figure 3F). We subjected the top 100 genes obtained from GEPIA2 to GO/KEGG analysis, Figures 3G,H suggest these gene enriched in “regulation of lymphocyte activation” and “phagocytosis” pathway.

### Correlation Between TMEM176B and Tumor Immune Infiltration

**FIGURE 4:**
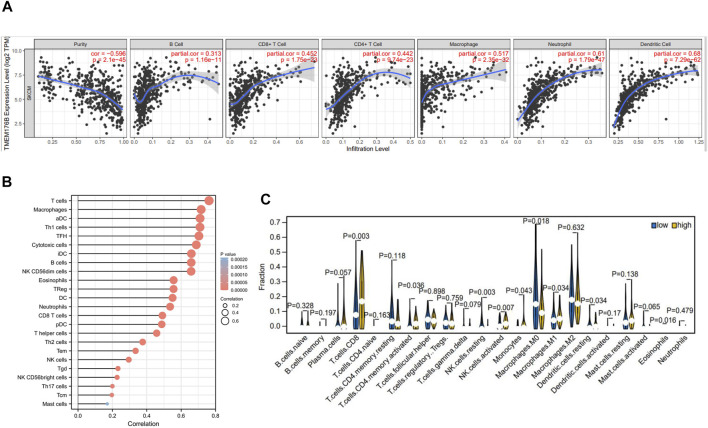
Correlation of *TMEM176B* expression and the level of immune infiltration in SKCM. **(A,B)** In SKCM, the infiltration levels of B cells, CD8^+^ T cells, CD4^+^ T cells, macrophages, neutrophils, dendritic cells and other immune cells with *TMEM176B* expression. *p* < 0.05 is considered significant. **(C)** The varied proportions of 22 subtypes of immune cells in high and low *TMEM176B* expression groups in tumor samples.

As can be seen from the above, *TMEM176B* was highly expressed in tumor tissues of SKCM and that higher expression correlated with better prognosis of SKCM patients. A variety of immune cells with different functions are present in the tumor microenvironment, including immunosuppressive cells and cells that promote tumor immunity (Quail and Joyce, 2013). As a result, it is necessary to continually explore the relationship between TMEM176B and the infiltration of immune cells. Data from TIMER suggests statistical significance between *TMEM176B* expression and immune cells, with B cells, CD4^+^ T cells, CD8^+^ T cells, macrophages, neutrophils, and dendritic cells were significantly positively correlated with the expression level of *TMEM176B* in SKCM (Figure 4A). *TMEM176B* expression levels, on the other hand, were significantly adversely associated with tumor purity (Figure 4A). We can also derive the association among *TMEM176B* expression and other immune cells from Figure 4B. Supplementary Figures S3A–C demonstrated correlation of *TMEM176B* expression and immune infiltration in different cancers of TCGA except SKCM. Among them, high expression of *TMEM176B* is associated with a higher degree of immune infiltration in most tumors. However, in LIHC, COAD, MESO and DLBC, the correlations were negative.

Using the CIBERSORT method, we investigated *TMEM176B* expression profiles to assess the level of 22 different immunocytes (Figure 4C). Our findings revealed that 9 kinds of immunocytes, including CD8^+^ T cells, activated CD4 memory T cells, resting and activated NK cells, Monocytes, Macrophages, resting dendritic cells and eosinophils were influenced by *TMEM176B* expression, among these cells CD8^+^ T cells and macrophages were significantly different between the *TMEM176B* high and low groups. The results of the high expression group compared to the low expression group showed that CD8^+^ T cells increased, whereas M0 Macrophages decreased. These findings imply that TMEM176B modulates patient survival via immunological infiltrative interactions in the tumor microenvironment. CD8^+^ cytotoxic T lymphocytes (CTLs) are essential in antitumor immunity. Therefore, the impact of TMEM176B on CD8^+^ T cell infiltration level and the prognosis of tumor patients deserves close attention.

### The Effects of *Tmem176b* on CD8^+^ T Cells in the TME

**FIGURE 5:**
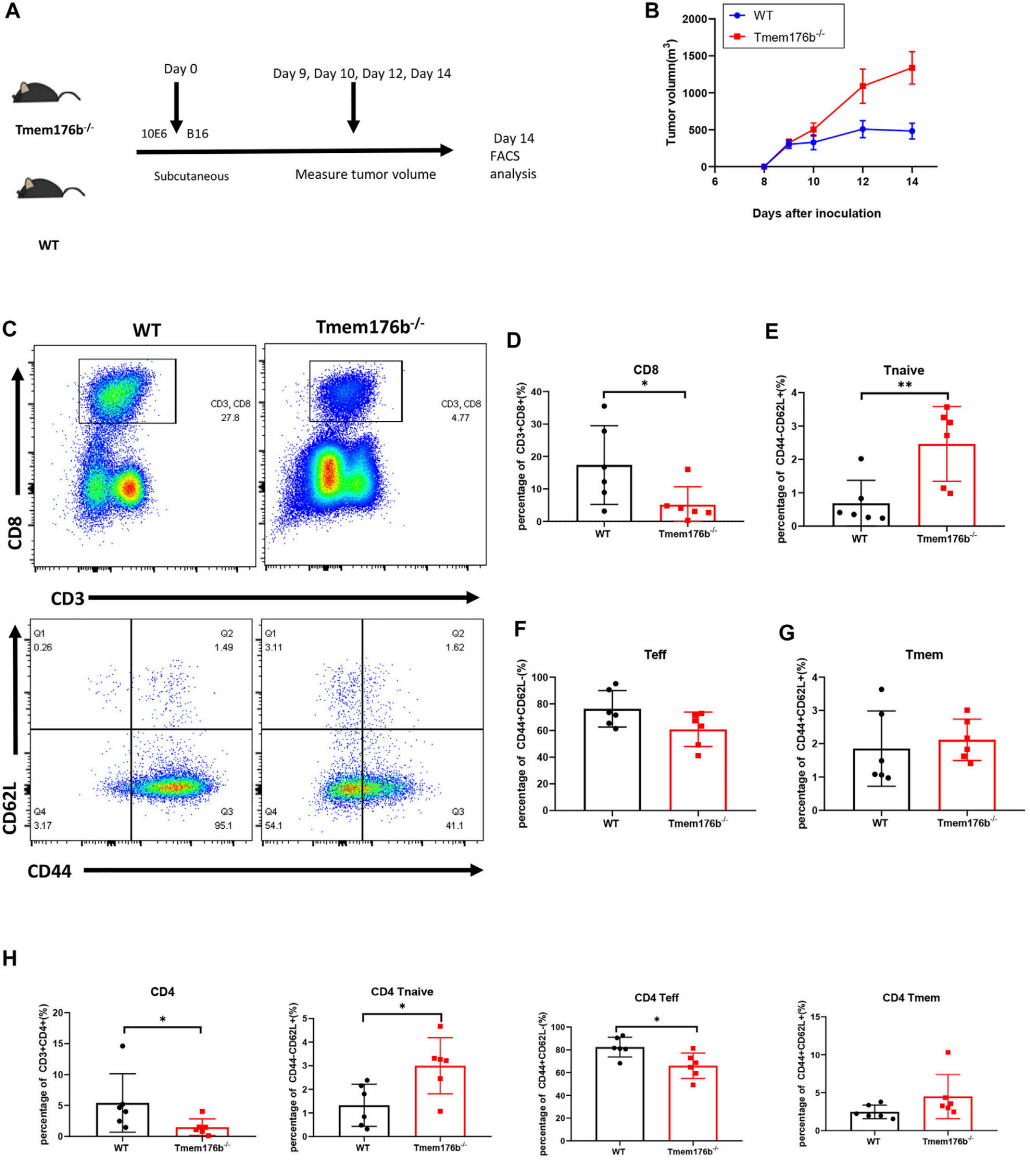
The regulatory effect of *Tmem176b* on T cells after tumor implantation. **(A)** Schematic diagram of mouse tumor model experiment. Six female mice per group. **(B)** Tumor growth curves of WT and *Tmem176b* ^−/−^ mice injected subcutaneously with 1 × 10^6^ B16-OVA melanoma cells (per group *n* = 6). **(C)** Flow cytometry of tumor-infiltrating lymphocytes TIL CD8^+^ T cells (top rows) and its subgroup (bottom rows) from WT and *Tmem176b* ^−/−^ mice at day 14 after subcutaneous injection with 1 × 10^6^ B16-OVA melanoma cells (*n* = 6). **(D–G)** Percentage of tumor-infiltrating CD3^+^ CD8^+^ T cells and their subpopulations Tnaive, Teff, Tmem from mice as in **(C)** (*n* = 6). **(H)** Percentage of tumor-infiltrating CD3^+^ CD4^+^ T cells and their subpopulations Tnaive, Teff, Tmem from mice as in **(C)** (*n* = 6). Each symbol represents a single mouse; data are mean SD from six independent samples. ns, not significant (two-tailed unpaired Student’s t test). *p < 0.05, **p < 0.01, and ***p < 0.001.

We would like to further study the regulatory relationship between TMEM176B and CD8^+^ T cells in the tumor microenvironment (TME). We investigated the role of *Tmem176b* in a mouse melanoma model and sought to continue to explore the role of *Tmem176b* in the regulation of CD8^+^ T cells in the tumor microenvironment. We constructed a mouse tumor model (Figure 5A) to figure out how *Tmem176b* regulates the differentiation, function, and immune infiltration levels of CD8^+^ T cells in the tumor microenvironment. After implanting the tumor subcutaneously for 14 days, we euthanized the mice for flow cytometry analysis. The tumor volume of mice following *Tmem176b* knockout was similarly greater and grew quicker than that of WT mice (*p* < 0.05) (Figure 5B).

Our findings revealed that the proportion of CD3^+^ CD8^+^ T cells in Tumor-infiltrating T lymphocytes (TILS), is dramatically reduced after *Tmem176b* knockout (Figures 5C,D), the same trend can be seen in CD3^+^ CD4^+^ T cells (Figure 5H). This downward trend was also found in peripheral blood, spleen, and lymph nodes, although we did not obtain statistical differences in all tissues (Supplementary Figure S4A), and this difference is more pronounced in CD8^+^ T cells, consistent with our previous results that higher expression of *TMEM176B* in SKCM was associated with higher levels of infiltration of immune cells (Figure 4). CD8^+^ T cells are subdivided into naive T cells (Tnaive), effector T cells (Teff) and memory T cells (Tmem) subsets. In our study, compared to wild type mice, more CD8^+^ Tnaive and less CD8^+^ Teff presented in tumor of *Tmem176b* ^−/−^ mice (Figures 5E,F), and the same change had also been observed in tumor-infiltrated CD4^+^ T cells (Figure 5H). However, Tmem remained unchanged between groups (Figures 5G,H). The influence of *Tmem176b* on the differentiation of T cells was not significant in other non-tumor organs (Supplementary Figures S4B,C). It suggested that T cell populations were normal in non-tumor organs and T cell homeostasis was altered in response to immunogenic stress exhibited by the facts that increased Tnaive cell number and decreased Teff cell number were presented in the local tumor of *Tmem176b* ^−/−^ mice.

**FIGURE 6:**
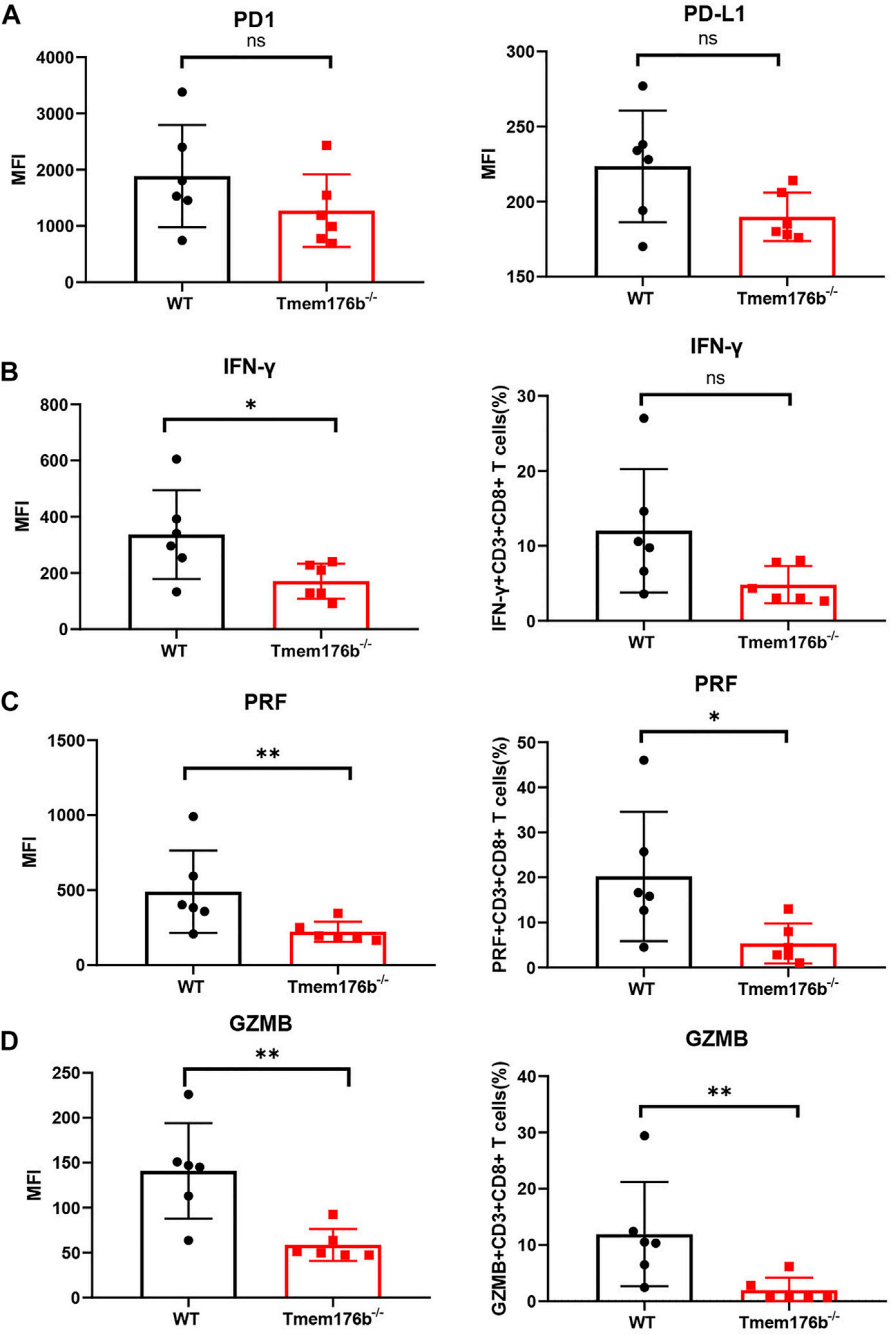
The regulatory effect of Tmem176b on T cells after tumor implantation. **(A)** The expression level of PD1 and PD-L1 of tumor-infiltrating CD8^+^ T lymphocytes (TIL CD8^+^ T cells) from WT and *Tmem176b* ^−/−^ mice (*n* = 6). **(B–D)** The MFI (left row) level of IFN-γ, PRF and GZMB of TIL CD8^+^ T cells, and frequency (right row) of IFN-γ^+^, PRF^+^ or GZMB^+^ (among TIL CD8^+^ T cells) from mice as in **(A)** (*n* = 6). Each symbol represents a single mouse; data are mean SD from six independent samples. ns, not significant (two-tailed unpaired Student’s t test). *p < 0.05, **p < 0.01, and ***p < 0.001.

We also looked at the expression of some immunosuppressive molecules following tumor transplantation. Comparing to WT mice, our findings revealed that the expression of multiple activation or effector function-related molecules were inhibited in tumor-infiltrated CD8^+^ T cells of *Tmem176b* ^−/−^ mice (Figure 6) although the trend was not consistent in blood, spleen or lymph node (Supplementary Figures S4D, S5). Our findings showed that after tumor implantation, knocking down *Tmem176b* has effect on T cell activation rather than anti-apoptosis, or proliferation (Supplementary Figure S5).

We could conclude from Figure 6 that the expression levels of cytotoxic molecules such as IFN-γ, PRF1, and GZMB in tumor-infiltrating CD8^+^ T cells was reduced following *Tmem176b* deletion, indicating that the function of tumor-infiltrating T cells was significantly compromised. It is the possible reason for the larger tumor size in *Tmem176b* knockout mice. To verify our results, we researched the correlation between the expression of *TMEM176B* and some molecules in SKCM. As shown in Supplementary Figure S6, the expression of *TMEM176B* was significantly positive related to the expression levels of PD-L1 (CD274), CTLA4, LAG3, GZMK, GZMB, GZMA, GZMM, PRF1, TNF and IFNG in SKCM. PD1 is a hallmark of T-cell exhaustion related to co-expression of other checkpoint molecules such as LAG-3 and TIM-3, as well as an activation marker for T-cells including antigen specific responses. The results of this part indicate that *Tmem176b* positively regulates the effector function and activation of tumor-infiltrating CD8^+^ T cells.

## Discussion

Immune cells play an essential role in tumor immunity, however, to date, there are few reports on the association of TMEM176B with immune cells. *Tmem176b* is predominantly expressed in BMDCs and cDC, and is involved in maintaining the immature state of DC (Condamine et al., 2010). In addition, strong expression of *Tmem176b* in a family of retinoic acid-related orphan receptor RORγt^+^ lymphocytes have been reported (Drujont et al., 2016; Lancien et al., 2021). Our research discovered a link between the expression of *TMEM176B* and the infiltration degree of immunocytes in cancers. *TMEM176B* was found to be substantially connected with the purity of most tumors in TIMER, as well as the infiltration of B cells, CD4^+^ T cells, CD8^+^ T cells, macrophages, neutrophils, and dendritic cells. In SKCM, our results indicated that *TMEM176B* was significantly and positively correlated with the infiltration of most immune cells. Robust infiltration of lymphocytes in melanoma correlates with a reduced risk of tumor growth (Galon et al., 2006).

Next, we used an animal model to identify the connection between *Tmem176b* and immune cell infiltration. After tumor implantation, we tested the effect of deleting *Tmem176b* on T lymphocyte development and function in diverse tissues and immunological organs. Lancien, M. et al. reported that *Tmem176a* and *Tmem176b* (*Tmem176a/b*) deficiency has no effect on tumor growth in MCA101-sOVA fibrosarcoma and B16-OVA melanoma (Lancien et al., 2021). However, our results showed that in comparison to wild-type mice, *Tmem176b* knockout mice had a greater volume of tumors. The study has (Segovia et al., 2019) reported that targeting TMEM176B controls tumor growth. The observed differences and inconsistency may due to the fact of tumor heterogeneity in which diverse roles of certain gene in different tumor models.

It was found that deletion of *Tmem176a* and *Tmem176b* does not affect CD8^+^ T cell responses but plays an irreplaceable role in the optimal initiation of initial CD4^+^ T cells by DCs (Lancien et al., 2021). Our results showed that the overall proportion of CD3^+^ CD4^+^ T and CD3^+^ CD8^+^ T cells in tissues was reduced, and tumor-infiltrating T lymphocytes (TIL CD4^+^ T, TIL CD8^+^ T) were significantly reduced in *Tmem176b* ^−/−^mice, which is consistent with our findings from public databases, where the higher the expression of *TMEM176B*, the higher the degree of T cell infiltration in SKCM. The reduced proportion of CD8^+^ T cells in *Tmem176b* knockout mouse could explain our finding of the accelerating progress and a poor prognosis in same mice model. Because high levels of CD8^+^ T cell infiltration have been shown to facilitate tumor treatment in tumors such as breast cancer and uroepithelial carcinoma (Sharma et al., 2007; Mahmoud et al., 2011).

In addition, we observed that the expression of some immunosuppressive molecules such as PD1, PD-L1 in *Tmem176b* knockout mice was lower than WT mice, and this downward trend was more significant at the level of PD-L1 expression. Although sometimes high PD-L1 levels predict suppression of anti-cancer immune responses upon binding to PD-1, PD-L1 expression is not necessarily equivalent to immune escape (Taube et al., 2014). A clinical study has shown that high PD-L1 expression on immune cells in uremic patients is associated with an increased probability of response to atezolizumab (An immune checkpoint blocker targeting PD-L1) (Rosenberg et al., 2016). Therefore, it is necessary to clarify that although high expression of immunosuppressive molecules or their ligands in tumors usually indicates the failure of the immune response that accompanies disease progression, they may also be a marker for the effectiveness of blockade therapies (Galluzzi et al., 2018). It has been explained that transcriptional regulation of PD-L1 may promote immune inflammation in the local tumor microenvironment, thus making tumors more sensitive to immune checkpoint blockade therapies (Gao et al., 2020). In addition, the increased expression of PD-1 in CD8^+^ T cells does not exactly indicate that the cells are in an exhaustion state, but also symbolize the activation of the cells (Wherry and Kurachi, 2015).

In the study, we found that, in comparison to wild-type animals, *Tmem176b* knockout lowered the expression of IFN-γ, PRF1 and GZMB in TIL CD8^+^ T cells, indicating that the function of tumor-infiltrating T cells was suppressed. As a result of that enhanced expression of IFN-γ and GZMB in the tumor microenvironment can achieve optimal tumor clearance (Lin et al., 2014). We analyzed the expression of *TMEM176B* in SKCM with the expression of some T cell-related genes to confirm our findings in the mouse tumor model, and we discovered that the expression of *TMEM176B* was significantly and positively linked with the expression of immunosuppressive molecules CD274 (PD-L1), CTLA4, LAG3, and cytotoxic molecules associated with T cell function TNF, PRF1, GZMA, GZMK, and GZMM, and most cytotoxic molecules, have been validated to act mainly as anti-tumoral and anti-infectious factors (Arias et al., 2017).

## Conclusion

Taken together, as one of the aggressive malignancies, the survival rate of melanoma varies depending on the stage of diagnosis. We discovered that *TMEM176B* expression was higher in SKCM than in normal tissue, and enhanced expression was linked to better OS. Also, TMEM176B was positively linked to immune cell infiltration in SKCM, which may explain that TMEM176B affects the prognosis of tumor patients. These findings lead us to hypothesize that TMEM176B can be employed as a diagnostic and prognostic marker. To validate our hypothesis, we developed melanoma model in *Tmem176b* knockout mice and revealed that T cell infiltration and effector function were significantly inhibited. It suggests that TMEM176B plays an essential role in anti-tumor function of CD8^+^ T cells, but the underlying mechanisms need to be further investigated.

## Data Availability

The datasets presented in this study can be found in online repositories. The names of the repository/repositories and accession number(s) can be found in the article/Supplementary Material.

## Supplementary Material

Supplementary Table 1 Clinical characteristics of the SKCM patients based on TCGA.

Supplementary Table 2 The number of cases used in each cancer types.

## Glossary

ACC: Adrenocortical carcinoma
BLCA: Bladder urothelial carcinoma
BRCA: Breast invasive carcinoma
CESC: Cervical squamous cell carcinoma and endocervical adenocarcinoma
CHOL: Cholangiocarcinoma
COAD: Colon adenocarcinoma
DLBC: Lymphoid neoplasm diffuse large B-cell lymphoma
ESCA: Esophageal carcinoma
GBM: Glioblastoma multiforme
GBMLGG: Glioma
HNSC: Head and neck squamous cell carcinoma
KICH: Kidney chromophobe
KIRC: Kidney renal clear cell carcinoma
KIRP: Kidney renal papillary cell carcinoma
LAML: Acute myeloid leukemia
LGG: Brain lower grade glioma
LIHC: Liver hepatocellular carcinoma
LUAD: Lung adenocarcinoma
LUSC: Lung squamous cell carcinoma
MESO: Mesothelioma
OV: Ovarian serous cystadenocarcinoma
PAAD: Pancreatic adenocarcinoma
PCPG: Pheochromocytoma and paraganglioma
PRAD: Prostate adenocarcinoma
READ: Rectum adenocarcinoma
SARC: Sarcoma
SKCM: Skin cutaneous melanoma
STAD: Stomach adenocarcinoma
TGCT: Testicular germ cell tumors
THCA: Thyroid carcinoma
THYM: Thymoma
UCEC: Uterine corpus endometrial carcinoma
UCS: Uterine carcinosarcoma
UVM: Uveal melanoma
