# Supplementary material for: A Potential Diagnostic and Prognostic Biomarker TMEM176B and Its Relationship With Immune Infiltration in Skin Cutaneous Melanoma
Source: Front Cell Dev Biol. 2022 Mar 23;10:859958. doi: 10.3389/fcell.2022.859958 (PMC8986129; doi:10.3389/fcell.2022.859958)
Supplement: Supplementary file 1 [file Table1.docx]

**Supplementary Table 1**

Clinical Characteristics of the SKCM Patients Based on TCGA

| Characteristic | Low expression of TMEM176B | High expression of TMEM176B | p |
| --- | --- | --- | --- |
| n | 234 | 234 |  |
| T stage, n (%) |  |  | < 0.001 |
| T1 | 15 (4.2%) | 26 (7.2%) |  |
| T2 | 37 (10.2%) | 41 (11.4%) |  |
| T3 | 41 (11.4%) | 49 (13.6%) |  |
| T4 | 99 (27.4%) | 53 (14.7%) |  |
| N stage, n (%) |  |  | 0.149 |
| N0 | 122 (29.7%) | 112 (27.3%) |  |
| N1 | 38 (9.2%) | 36 (8.8%) |  |
| N2 | 19 (4.6%) | 30 (7.3%) |  |
| N3 | 21 (5.1%) | 33 (8%) |  |
| M stage, n (%) |  |  | 1.000 |
| M0 | 207 (47%) | 209 (47.5%) |  |
| M1 | 12 (2.7%) | 12 (2.7%) |  |
| Pathologic stage, n (%) |  |  | 0.002 |
| Stage I | 32 (7.8%) | 44 (10.8%) |  |
| Stage II | 89 (21.8%) | 51 (12.5%) |  |
| Stage III | 75 (18.3%) | 95 (23.2%) |  |
| Stage IV | 11 (2.7%) | 12 (2.9%) |  |
| Radiation therapy, n (%) |  |  | 0.026 |
| No | 198 (43%) | 183 (39.7%) |  |
| Yes | 30 (6.5%) | 50 (10.8%) |  |
| Gender, n (%) |  |  | 0.087 |
| Female | 80 (17.1%) | 99 (21.2%) |  |
| Male | 154 (32.9%) | 135 (28.8%) |  |
| Race, n (%) |  |  | 0.771 |
| Asian | 7 (1.5%) | 5 (1.1%) |  |
| Black or African American | 0 (0%) | 1 (0.2%) |  |
| White | 223 (48.7%) | 222 (48.5%) |  |
| Age, n (%) |  |  | 0.113 |
| <=60 | 115 (25%) | 134 (29.1%) |  |
| >60 | 114 (24.8%) | 97 (21.1%) |  |
| Weight, n (%) |  |  | 0.244 |
| <=70 | 47 (18.3%) | 30 (11.7%) |  |
| >70 | 94 (36.6%) | 86 (33.5%) |  |
| Age, median (IQR) | 60 (49, 71) | 57 (47, 70) | 0.143 |
