# Supplementary figures and images for: A Potential Diagnostic and Prognostic Biomarker TMEM176B and Its Relationship With Immune Infiltration in Skin Cutaneous Melanoma
Source: Front Cell Dev Biol. 2022 Mar 23;10:859958. doi: 10.3389/fcell.2022.859958 (PMC8986129; doi:10.3389/fcell.2022.859958)

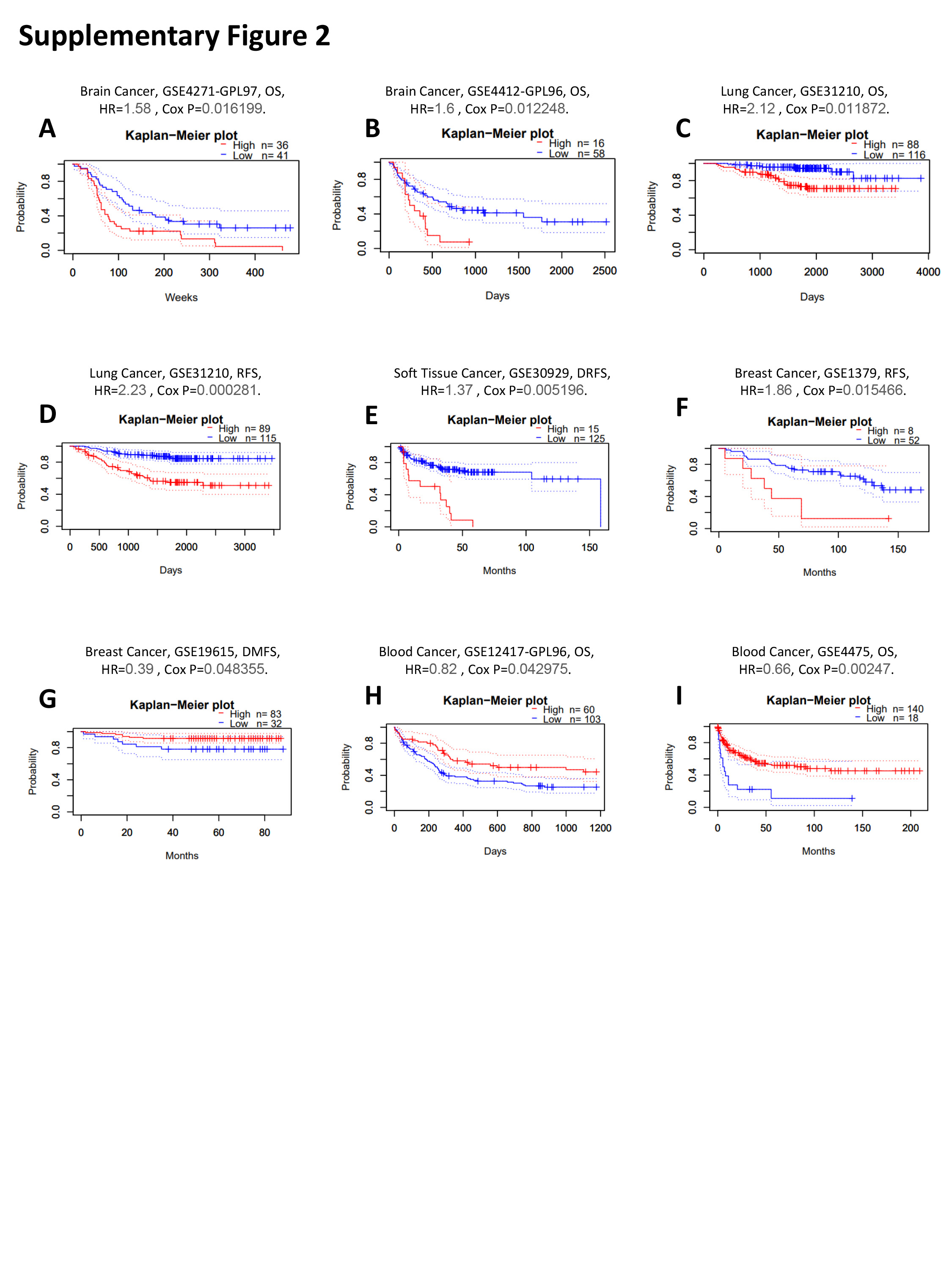

Supplement: Supplementary file 2 [file DataSheet1.ZIP › supplementary figure 2.jpg]

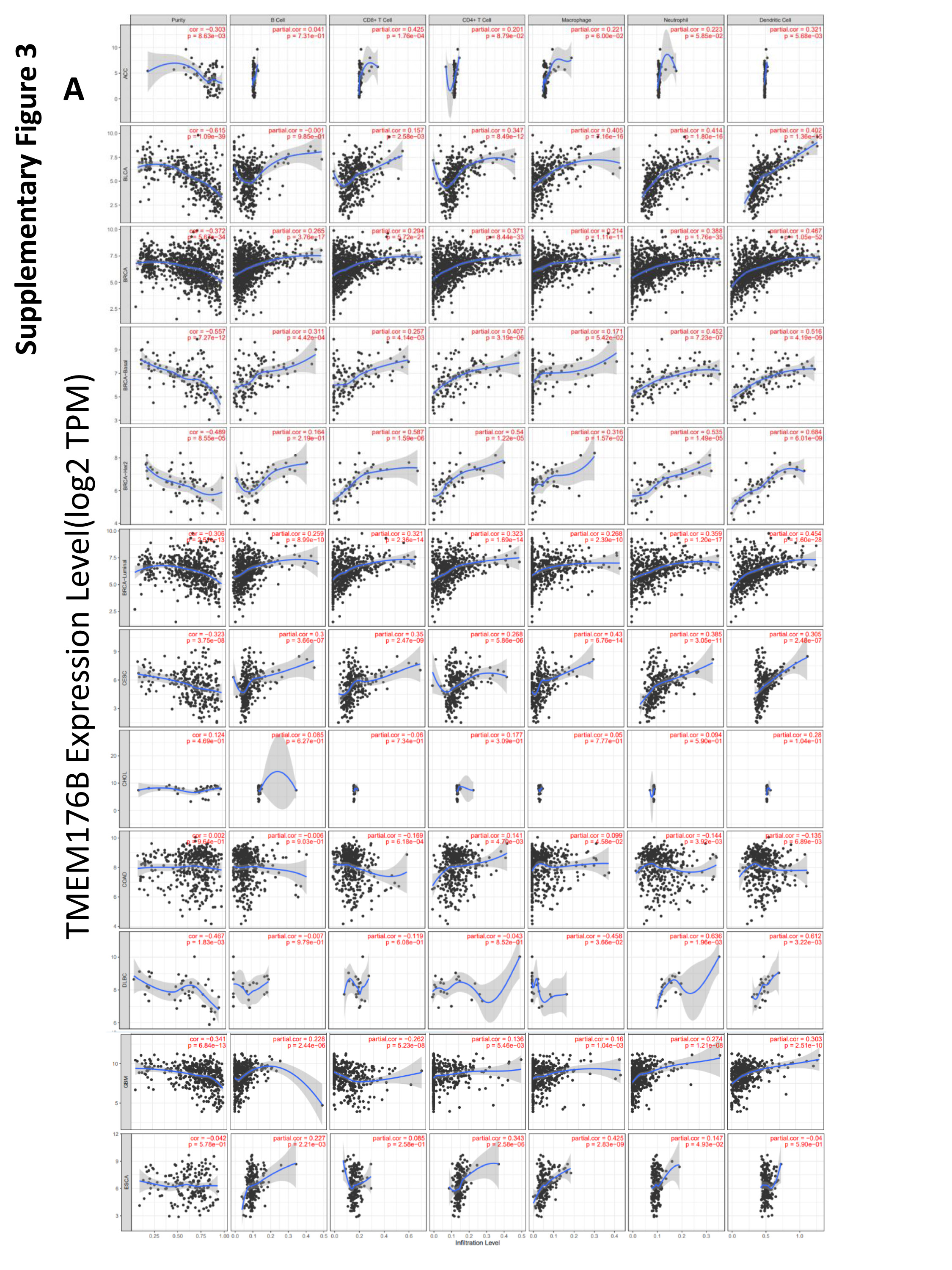

Supplement: Supplementary file 2 [file DataSheet1.ZIP › supplementary figure 3-A.jpg]

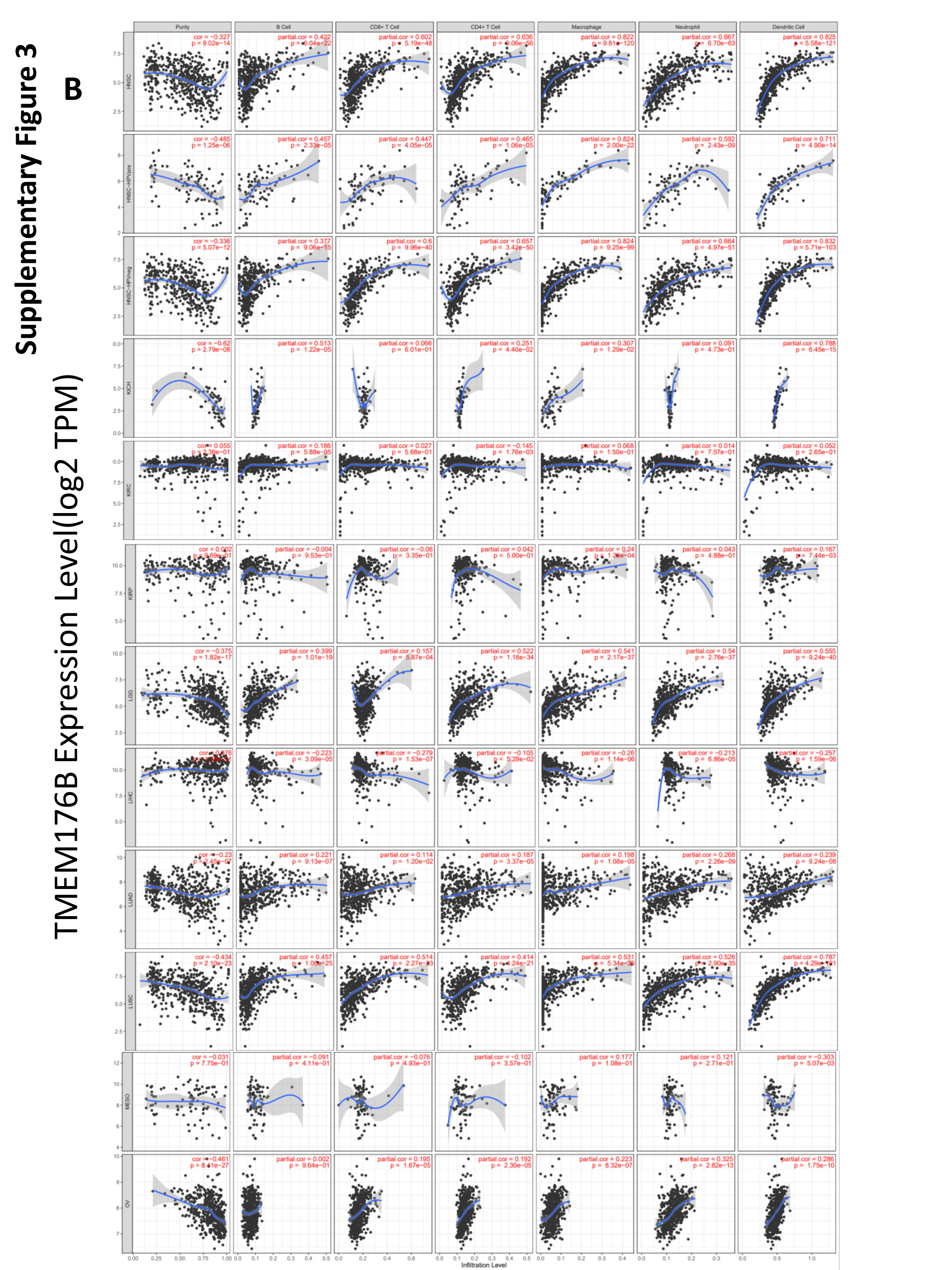

Supplement: Supplementary file 2 [file DataSheet1.ZIP › supplementary figure 3-B.jpg]

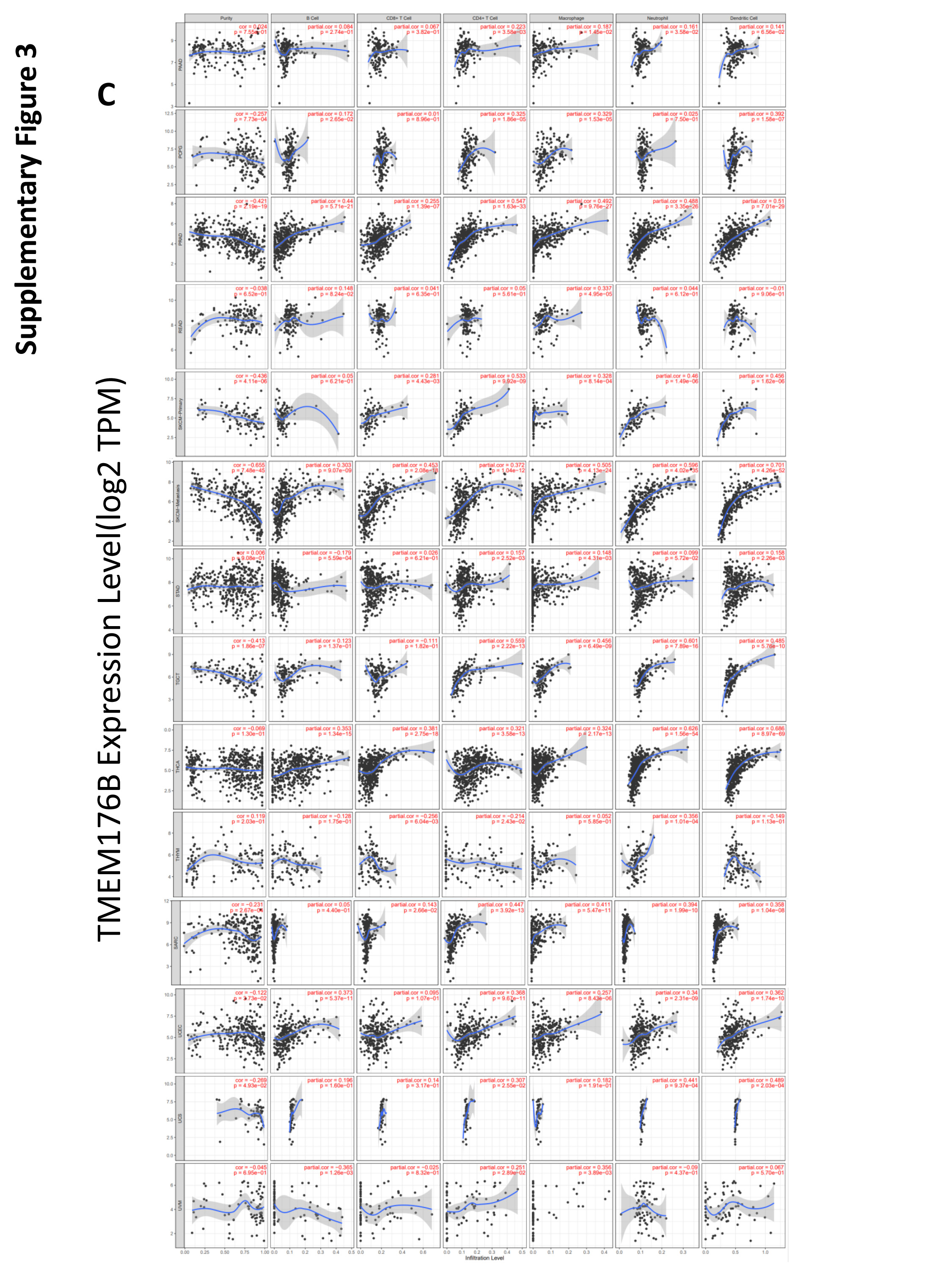

Supplement: Supplementary file 2 [file DataSheet1.ZIP › supplementary figure 3-C.jpg]

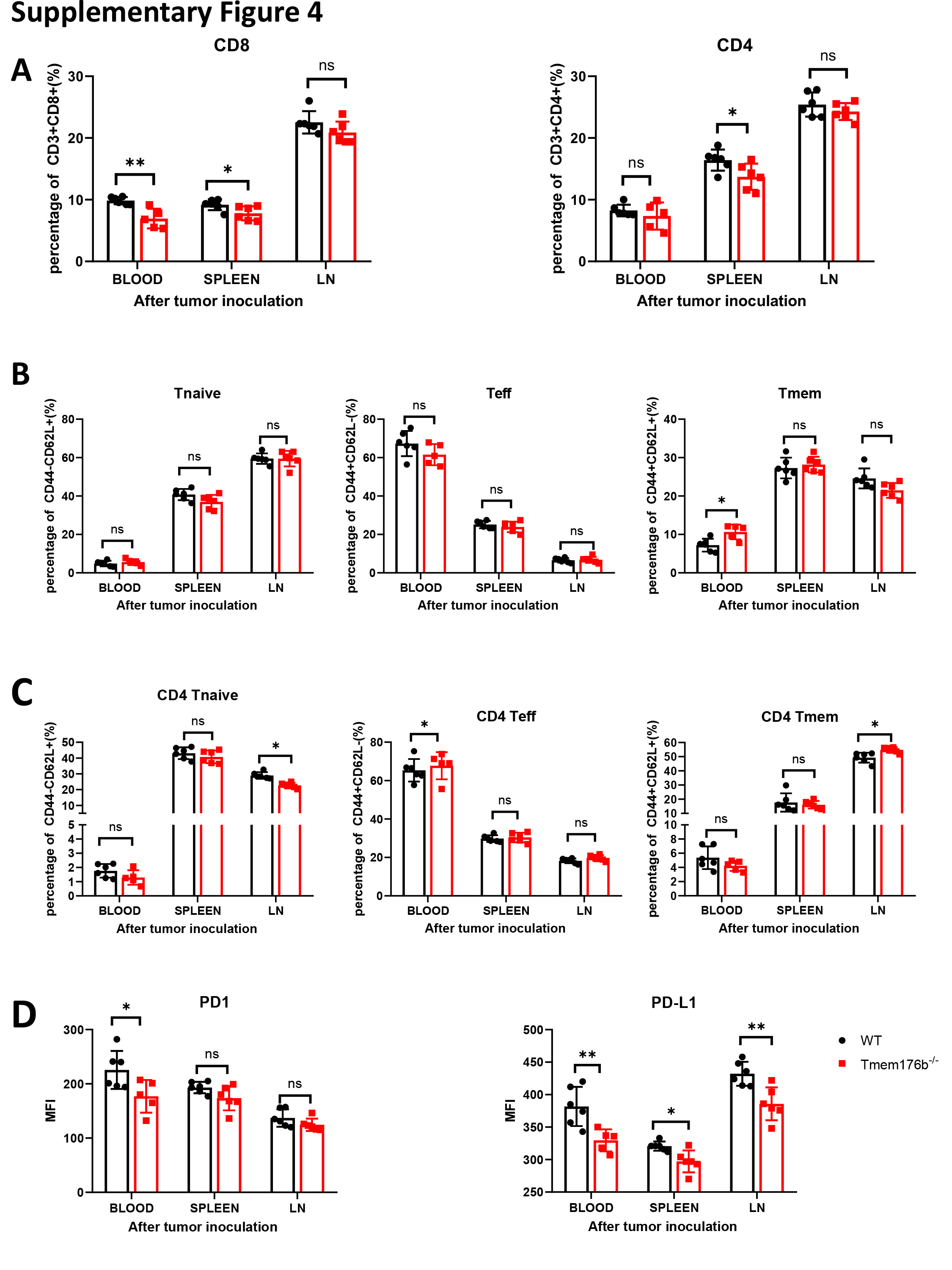

Supplement: Supplementary file 2 [file DataSheet1.ZIP › supplementary figure 4.jpg]

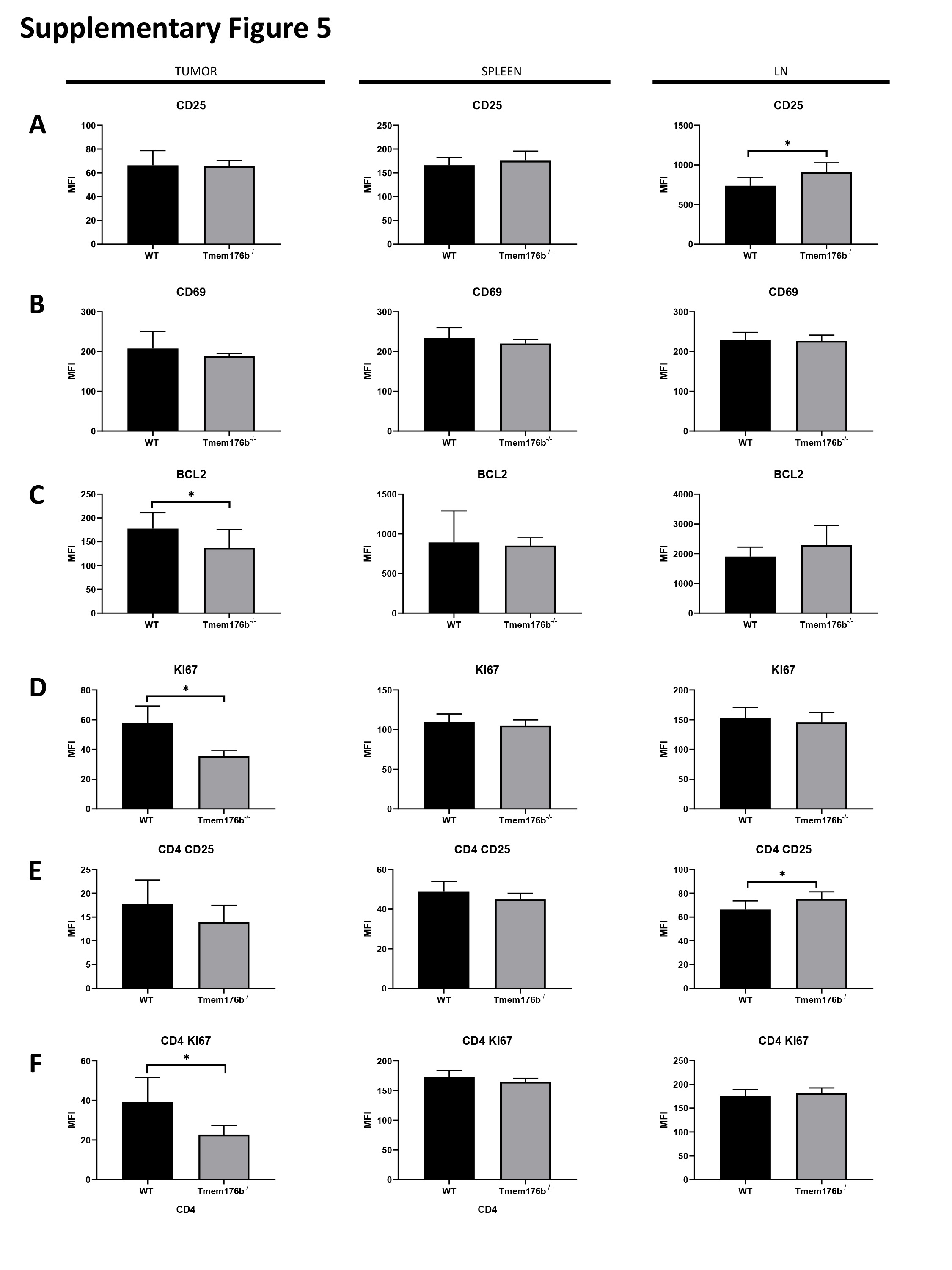

Supplement: Supplementary file 2 [file DataSheet1.ZIP › supplementary figure 5.jpg]

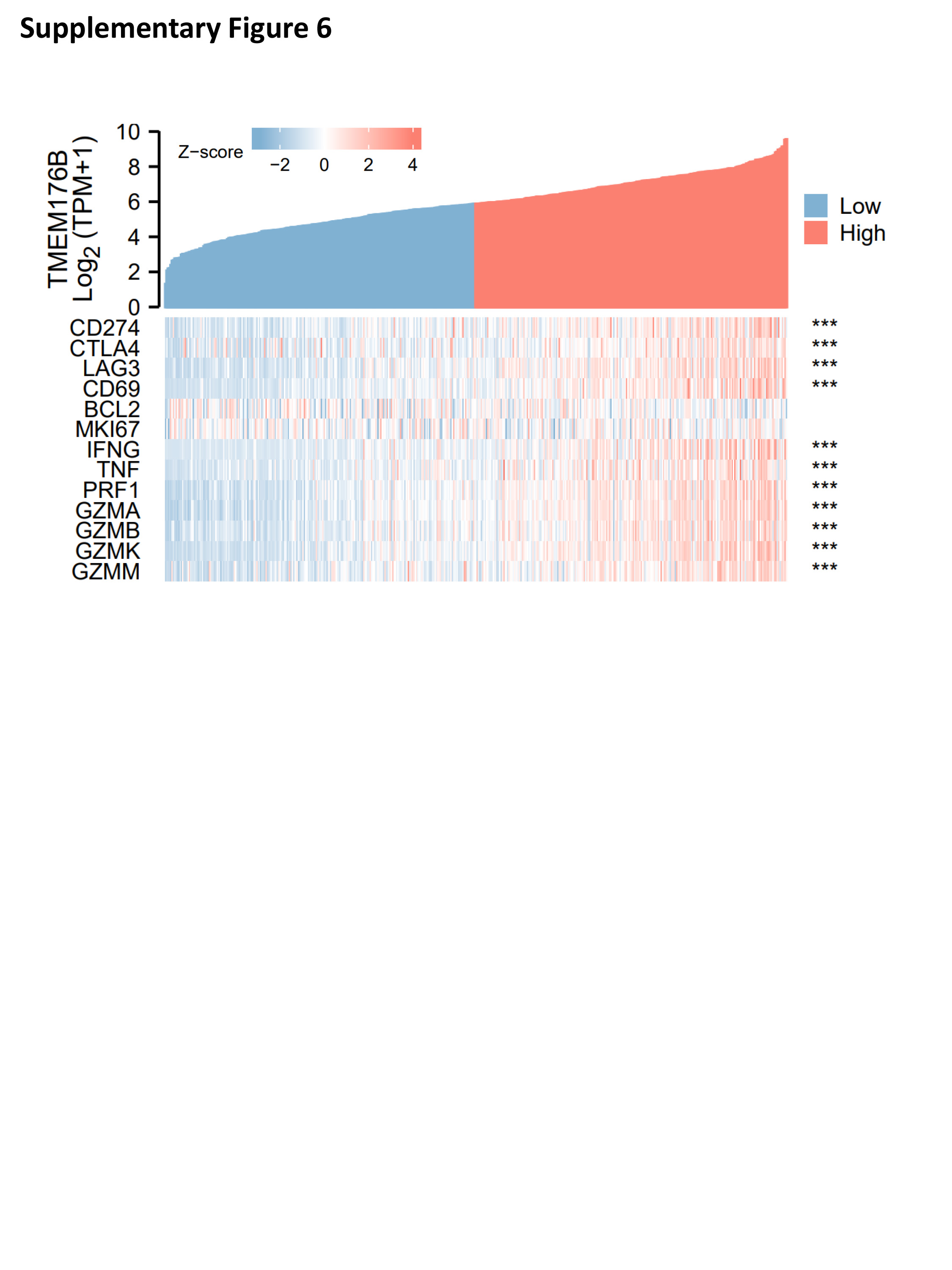

Supplement: Supplementary file 2 [file DataSheet1.ZIP › supplementary figure 6.jpg]

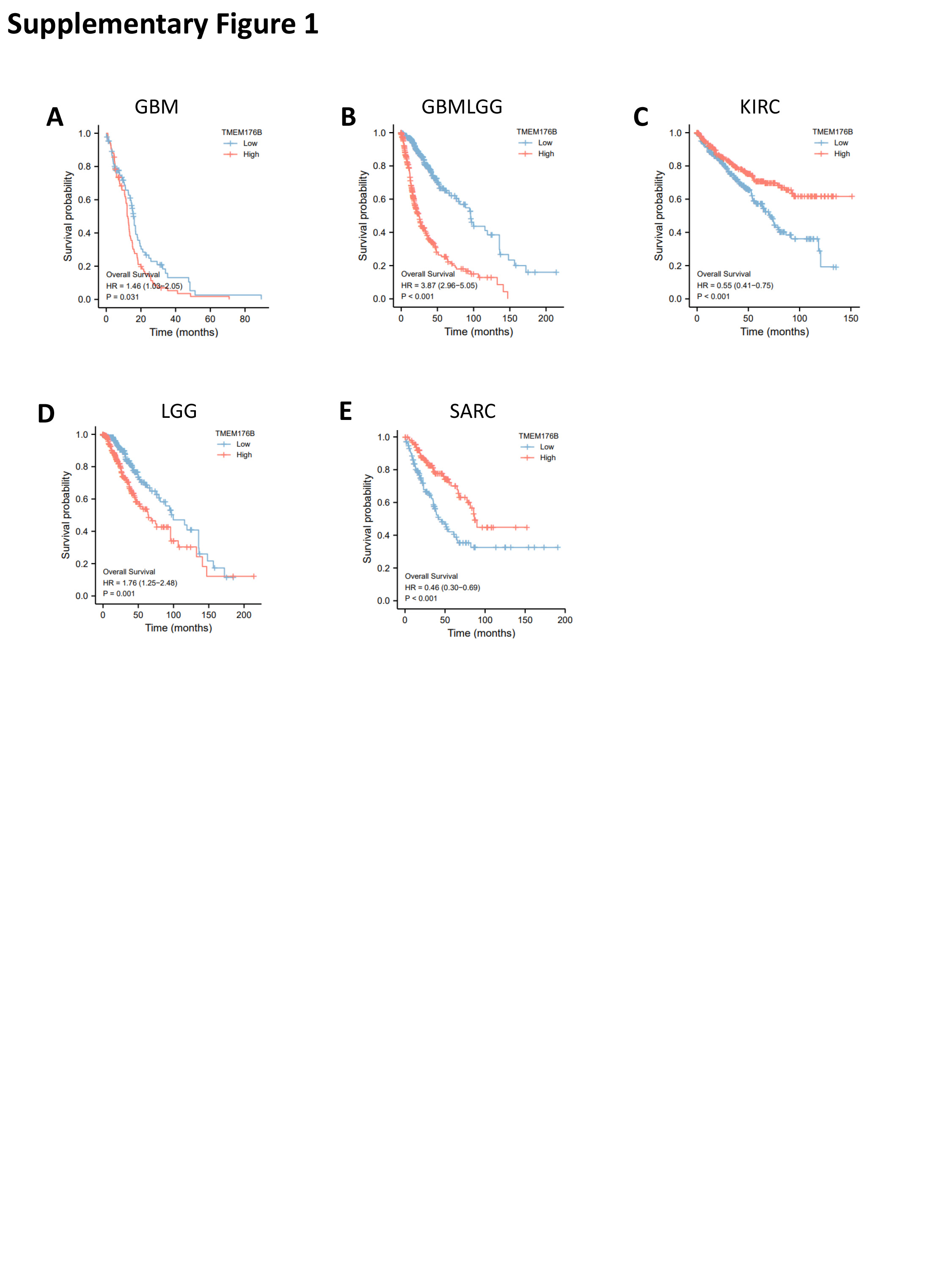

Supplement: Supplementary file 2 [file DataSheet1.ZIP › supplementary figure 1.jpg]
