# Supplementary material for: A Potential Diagnostic and Prognostic Biomarker TMEM176B and Its Relationship With Immune Infiltration in Skin Cutaneous Melanoma
Source: Front Cell Dev Biol. 2022 Mar 23;10:859958. doi: 10.3389/fcell.2022.859958 (PMC8986129; doi:10.3389/fcell.2022.859958)
Supplement: Supplementary file 3 [file Table2.docx]

**Supplementary Table 2**

The number of cases used in each cancer types.

| Group | Normal | Tumor |  |
| --- | --- | --- | --- |
| ACC | 128 | 77 |  |
| BLCA | 28 | 407 |  |
| BRCA | 292 | 1099 |  |
| CESC | 13 | 306 |  |
| CHOL | 9 | 36 |  |
| COAD | 349 | 290 |  |
| DLBC | 444 | 47 |  |
| ESCA | 666 | 182 |  |
| GBM | 1157 | 166 |  |
| HNSC | 44 | 520 |  |
| KICH | 53 | 66 |  |
| KIRC | 100 | 531 |  |
| KIRP | 60 | 289 |  |
| LAML | 70 | 173 |  |
| LGG | 1152 | 523 |  |
| LIHC | 160 | 371 |  |
| LUAD | 347 | 515 |  |
| LUSC | 338 | 498 |  |
| MESO | NA | 87 |  |
| OV | 88 | 427 |  |
| PAAD | 171 | 179 |  |
| PCPG | 3 | 182 |  |
| PRAD | 152 | 496 |  |
| READ | 318 | 93 |  |
| SARC | 2 | 262 |  |
| SKCM | 813 | 469 |  |
| STAD | 210 | 414 |  |
| TGCT | 165 | 154 |  |
| THCA | 338 | 512 |  |
| THYM | 446 | 119 |  |
| UCEC | 101 | 181 |  |
| UCS | 78 | 57 |  |
| UVM | NA | 79 |  |
